# Analysis of enzymatic digestion pattern of two open reading frames of Varciella–Zoster genome from Kuwaiti patients using the RFLP technique

**Published:** 2012-12

**Authors:** Qasem JA, MA Al-Fadhli, MA Saraya, J Thomas

**Affiliations:** 1 Department of Applied Medical Sciences, College of Health Sciences, Public Authority for Applied Education and Training-PAAET, Kuwait; 2 Department of Medicine, Infectious Disease Hospital, Ministry of Health, Kuwait

**Keywords:** Varicella, RFLP, Genotyping, Restriction enzymes, Kuwait

## Abstract

**Background and Objectives:**

Varicella–Zoster virus (VZV) is a human herpes virus that usually attacks young children and commonly causes chicken pox (Varicella). Following primary infection, a lifelong latent infection is established. The virus often reactivates during adulthood or senesces to cause shingles (Zoster). Little is known regarding the genotypes of Varicella in Kuwait. The aim of this study was to genotype Varicella samples collected from patients in Kuwait.

**Materials and Methods:**

Samples from 60 cases of chicken pox were typed. The DNA extraction was performed using the commercially available DNA extraction kit. Two sets of oligonucleotide primers were used to amplify the intervening sequences with polymerase chain reaction to identify VZV DNA in clinical samples. The *Bgl*I and *Pst*I endonucleases were used to digest. The PCR amplicons for PCR-RFLP typing.

**Results:**

Relatively consistent restriction enzyme digestion profiles for different VZV strains were observed. Limited genetic differences between VZV samples were found. Three VZV strains were identified (A, B and C) with type B representing 86.6%, type A 11.7% and type C being 1.7%. We found that distinct restriction fragment length polymorphism isolates from the same origin or nationality were very similar.

**Conclusion:**

Varicella strains with cutting sites for both enzyme *Pst*I and *Bgl*I (typeB) were more prevalent. Molecular amplification of viral DNA by PCR and restriction digestion could be used for VZV typing as an alternative method to serological assays.

## INTRODUCTION

Varicella-Zoster is a human herpes virus, a member of the subfamily Alphaheresvirinae within the family of Herpesviridae, commonly causing chicken pox (varicella), in young children. Following primary infection, a lifelong latent infection is established, and the virus often reactivates in adulthood or senesces to cause shingles (Zoster) (1).

Early efforts in VZV typing used DNA restriction fragment length polymorphism (RFLP) analysis (2–4), an approach that confirmed the identity of the VZV strain that caused varicella on primary infection and later reactivated to cause zoster. Relatively consistent restriction enzyme digestion profiles for different VZV strains have been observed, providing the first evidence that VZV has a highly conserved genome. Intra-strain variation in restriction enzyme fragment profiles among wild-type VZV isolates was observed by Straus *et al*. (5). However, the most prominent differences were linked to variation in the number and composition of VZV genome repeat elements (6, 7).

Varicella-Zoster virus (VZV) causes chicken pox infection in susceptible individuals, following which the virus remains latent in the dorsal root ganglia until reactivation to cause zoster in about 25% of the population. There is apparently little variation in this virus, and one serotype appears to be responsible for VZV disease worldwide (8). Limited genetic differences between VZV isolates have been reported, and restriction analysis of American and Japanese wild-type viruses have identified distinct American and Japanese restriction fragment length polymorphisms (9–11). VZV isolates from the same geographical area have been reported to be very similar (5).

Recent reports from the United States and the United Kingdom suggest that the epidemiology of VZV infection may be changing. In both countries, the incidence of chicken pox in adults (>15 year old) has risen over the past 20 to 30 years (8, 12).

Herpes zoster (shingles) is caused by recurrent varicella zoster virus (VZV) infection from a latent state. About 10% of humans are affected by shingles during their lifetimes (13). Transmission from zoster can reintroduce strains that were circulating decades earlier and, as such, contributes to the genetic stability of VZV (13).

The epidemiology of VZV infection varies geographically. Varicella displays a marked seasonality (peaking in late winter or spring) in temperate climates, and infection is nearly ubiquitous by the age of 20 years. For reasons that still remain unclear, seasonality does not occur in tropical countries, and a larger proportion of people enter adulthood uninfected by VZV (14, 15).

Several techniques have been reported for genotyping VZV strains, of these techniques genotyping using DNA sequence variation for the detection of a single nucleotide polymorphisms (SNPs) (16, 17) and phylogenetic alignment of nucleotide polymorphisms (18). Early VZV typing efforts relied primarily on DNA restriction fragment length polymorphism (RFLP) analysis, an approach that first demonstrated the high degree of sequence conservation among VZV strains. Nevertheless, some intra-strain variation among wild-type isolates of VZV was also observed (19, 20). RFLP has also been used to distinguish mostly the wild-type VZV isolates from Oka vaccine preparations, using single nucleotide polymorphisms (SNPs) located in open reading frame 38 (ORF38; a *Pst*I site present in many wild-type strains) and ORF54 (a *Bgl*I site found in Oka vaccine preparations) (21, 22).

On the basis of the analysis of single nucleotide polymorphisms (SNP) at least three or four geographically distinct genotypes have been described (17). Several VZV genotyping schemes have been developed, and each proposes a different classification for genotyping. Loparev et al. reported that by the analysis of a short region in ORF22 of VZV genome, three major genotypes can be distinguished, J (Japanese), M (Mosaic) and E (European). The genotype M was found to be further subdivided in to four types (M1, M2, M3 and M4) (17).

Recent phylogenetic analyses of partial and whole VZV genome sequences suggest that VZV genomic variation can be categorized into specific genotypes and probable recombinant viruses (23, 24). In the past, several studies had demonstrated the superiority of detection of VZV antigens by immunologic techniques. More recently, the molecular amplification of viral DNA by PCR assays has been compared with the serologic assays for immunoglobulin G or A class antibodies or the cultivation of this virus in cell cultures (25–28). Little information is known for the genotypes of Varicella from Kuwait. The aim of this study was to genotype Varicella samples collected from patients in the State of Kuwait.

## MATERIALS AND METHODS

### Clinical specimens

Sixty clinical specimens of vesicular dried scabs were collected from patients visiting the Infectious Disease Hospital (IDH), which is a tertiary care hospital located in Al-Sabah health district within the Al-Asimah governorate (capital governorate) of Kuwait, for examination by the Physicians specialized in infectious diseases between January, 2010 and December, 2010. These patients of different nationalities came from a variety of geographic locations in the State of Kuwait. To the extent possible, the age, sex and ethnic origin of the patients were ascertained. Clinical characteristics of varicella patients were reviewed and defined as vesicular eruption with generalized onset and no dermal distribution.

Oka vaccine viral DNA was prepared from a commercial lot of Varilrix (GlaxoSmithKline Biologicals, Belgium). Varilrix is a lyophilized preparation of the live attenuated Oka strain of varicella-zoster virus, obtained by propagation of the virus in MRC5 human diploid cell culture.

The study was approved by the Ethical Committee of the Ministry of Health and College of Health Sciences, Kuwait. This work was carried out in accordance with the code of ethics of the World Medical Association (Declaration of Helsinki for experiments involving humans). Each subject was interviewed and a signed informed consent was obtained.

### Amplification of target DNA

DNA extraction was made with the QIAmp DNA Mini Kit (Qiagen, Germany) according to the manufacturer's protocol. Two rounds of PCR were carried out using 5 µl of the extracted DNA in a total of 100 µl. All samples were initially characterized by the use of conventional PCR targeting VZV-specific sequences in ORF38 and ORF54, amplified, and then followed by restriction enzyme analysis. Two sets of primer pairs of 20 bp in length, were selected from published literature (21) and synthesized by Genosys Biotechnologies, UK.Primers Nla (GGAACCCCTGCACCATTAAA); Fok (TCCCTTCATGCCCGTTACAT) with expected product of 222 base pairs in size and primers Pst A (TTGAA CAATCACGAACCGTT); Pst B (CGGGTGAACCGTATTCTG AG) with expected product of 350 base pairs in size.

The PCR reaction mixture contained: 10 mM Tris-HCL, pH 8.3, 50 mM KCL, 1.5 mM MgCl_2_, 0.001% gelatin, 4% dimethyl sulfoxide (DMSO), 200 mM of each dNTP, 02 µM of each primer and 2.5 units of AmpliTaq DNA polymerase (Perkin–Emer, Branchburg, NJ (USA). The targeted DNA was amplified for 30 PCR cycles, each cycle consisted of denaturation for 1 minute at 94°C, annealing for 1 min at 52°C and elongation for 1 min at 72°C. A pre-denaturing step at 94°C for 3 minutes was included with each of the amplification.

### RFLP analysis

RFLP analysis was carried out after digestion of the PCR amplified DNA with the commercially available restriction endonucleases, *Bgl*I, *Pst*I, according the manufacturer's instructions. The digested DNA was analyzed by gel electrophoresis on 2% Ultra-pure agarose gel (GIBCO-BRL life Technologies, Paisley, UK), at 7–10 V/cm for 1–1.5 h in TAE buffer containing 0.5µg/ml ethidium bromide. A molecular size marker (100 bp ladder, BRL) was included in each gel.

## RESULTS

**Fig. 1:**
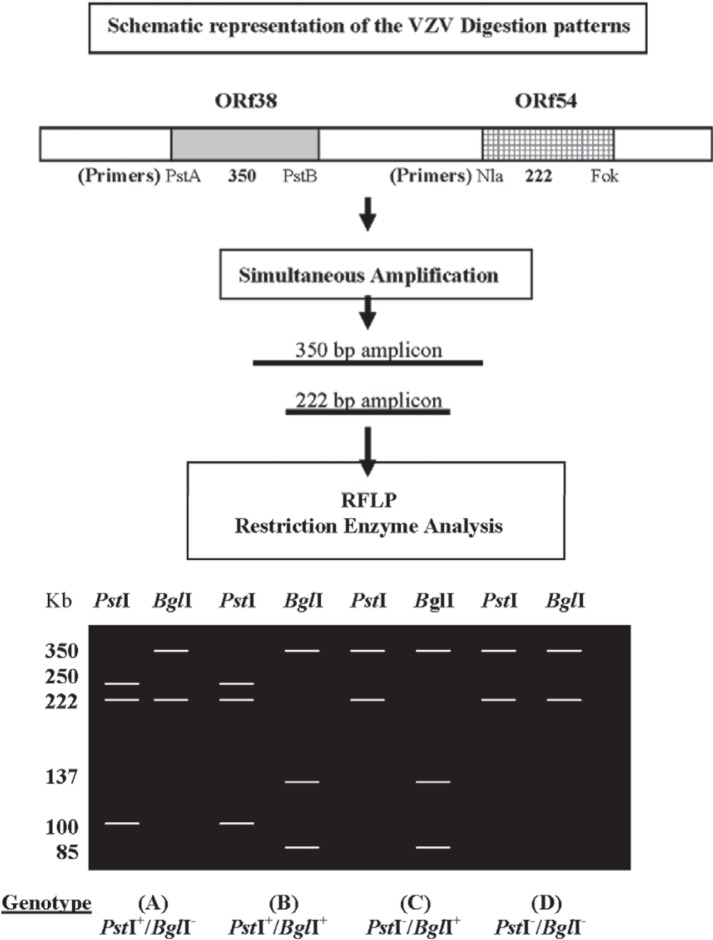
A schematic diagram indicating the PCR amplification of two ORf's fragments using two primersset. Ethidum bromide agarose gel indicating different genotypes corresponding to restriction enzyme (*Pst*I and *Bgl*I) digestion. The 350 and 225 base pair amplicons were incubated with *Ps*tI and *Bgl*I restriction enzymes for digestion. The product of digestion is represented by agarosegel showing sizes of digestion products. The capital letters (A, B, C, D) in the lower portion of the figure represent the genotypes.

Sixty DNA preparations from chicken pox cases were typed using RFLP method targeting (ORF 54-ORF 38). For the ORF 54- ORF 38 method, 222-bp and 350-bp amplicons were produced and digested with *Pst*I and *Bgl*I restriction enzymes, respectively (Fig. 1 and Table 1). Three of four possible genotypes were detected: 7 specimens were identified as wild-type *Pst*I^+^/*Bgl*I^−^ (i.e., possessing and lacking a *Pst*I and a *Bgl*I restriction site, respectively, and 52 specimens were identified as wild-type *Pst*I^+^/ *Bgl*I^+^ Additionally, one DNA was typed as Oka vaccine strain (*Pst*I^*−*^ */ Bgl*I^+^). The fourth possible genotype (*Pst*I^−^^/^ *Bgl*I^−^) was not identified in this study.

**Table 1:** Prevalence of HSV genotype between samples of patients in Kuwait using Restriction Fragment Length polymorphism (RFLP) typing method.

Original country of patient	Total	Percentage %	No of total with site for		Genotype			
			*Pst*I	*Bgl*I	A *Pst*I^+^/*Bgl*I^−^	B *Pst*I^+^/*Bgl*I^+^	C *Pst*I^−^/*Bgl*I^+^	D *Pst*I^−^/*Bgl*I^−^
Kuwait	4	6.7%	4	3	1 (25%)	3 (75%)	0	0
Saudi	1	1.7%	1	1	0	1 (100%)	0	0
India	31	51.7.%	31	27	4 (13%)	27 (87%)	0	0
Sri Lanka	11	18.3%	11	9	2 (18%)	9 (82%)	0	0
Bangladesh	5	8.3%	5	5	0	5 (100%)	0	0
Philippine	7	11.7%	6	7	0	6 (86%)	1 (14%)	0
Ethiopia	1	1.7%	1	1	0	1 (100%)	0	0
Total	60				7 (11.66%)	52(86.6%)	1(1.66%)	0

The two 20-bp oligonucleotides (Nla/Fok) flanking the *BgI*l site produced an amplicon of 222 bp in wild-type and an Oka DNA. The amplification product of wild-type DNA lacked a *Bgl*I site; therefore, the DNA didn't cleave after incubation with *BgI*I. However, the amplification product of vaccine-type DNA was cleaved with *Bgl*I, resulting in two fragments of 137 and 85 bp (Fig. 1).

The second primer pair (Pst A/B) amplified a 350-bp fragment asymmetrically brackets a *Pst*I site, present in all wild-type isolates that we had analyzed, including the *Bgl*I^+^ WT strain, but that was absent from Oka DNA (Fig. 1).

**Fig. 2:**
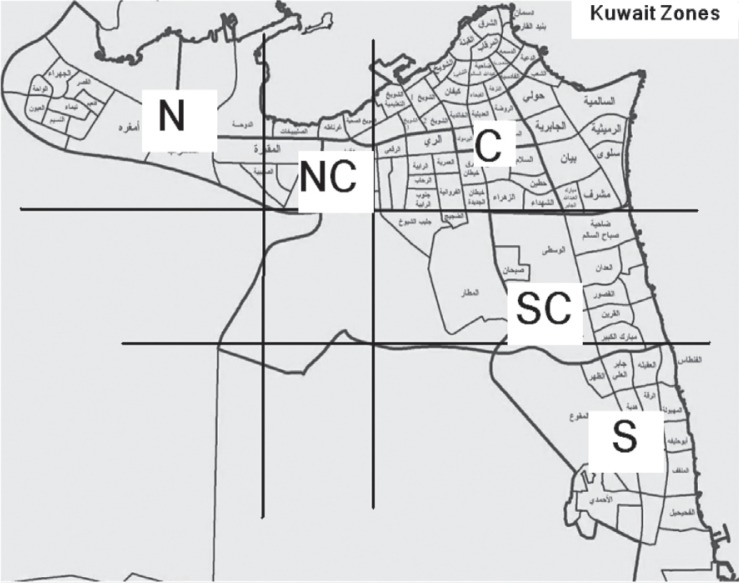
Map showing geographic distribution of the resident locations of patients coming to Infectious Diseases Hospital, Kuwait, as prepared from the information provided by Civil ID of each patient. North (N = 10/B), North Central (NC = 11/B), Central (20/B), South Central (SC = 5/B), South (S = 7/A; 6/B; 1/C).

A schematic representation of the digestion patterns of wild-type, *Bgl* ^+^ WT, and Oka DNAs is shown in Fig. 1. The 222-bp amplification product from wild-type isolates was not cleaved by *Bgl*I, whereas its 350-bp amplification product was cleaved by *Pst*I. In contrast, the 222- and 350-bp amplification products of *BgI*I^+^ WT isolates were cleaved by *BgI*l and *Pst*I, respectively. The 222-bp Oka amplification product was cleaved by *Bgl*I, whereas its 350-bp amplification product was not cleaved by *Pst*I.

RFLP analysis indicated that *Pst*I^+^/*Bgl*I^-^ viruses represent only 11.66% of the samples collected in the State of Kuwait (Table 1). In a representative subset of 52 samples presented in Table 1, 86.6% were determined to have the *Pst*I^+^/*Bgl*I^+^ profile. This genotype (B) was also the most commonly isolated genotype in Indian (87%), Sir Lankan (82%), Bangladeshi (100%), Philipino (86%), Ethiopian (100%), Saudi Arabian (100%), and Kuwaiti (75%) patients. In contrast, the *Pst*I^+^/*Bgl*I^−^ genotype represented as the lowest in the Kuwaiti samples (11.66%). As shown in Table 1, one specimen collected from Kuwaiti, four from Indian, and one from Sri Lankan were found to be *Pst*I^+^/*Bgl*I^−^ type.

Seven strains of type A were found to be *Pst*I^+^/ *Bgl*I^-^, and 52 strains were *Pst*I^+^/ *Bgl*I^+^, in contrast to the Oka reference control, which carried the *Pst*I^-^/ *Bgl*I^+^ profile typical of C strains (Table 1). Only one of the tested patient samples gave restriction pattern similar to Oka strain, the strain C (Table 1). Obviously, 60 specimens from diverse geographic locations in the State of Kuwait and different nationalities were evaluated in this study.

The central zone of the State of Kuwait had the highest (20) number of VZV cases (33.3%), the south zone had the most variable genotype 7 (11.7% A), 6 (10% B), and 1 (1.7% C). The north and north central regions had similar frequencies of genotypes B (Table 2).

**Table 2:** Frequency of different genotypes of VZV, distributed according to geographical locations and gender of patients in Kuwait.

VZV Genotype	Geographical locations					Gender	
	N	NC	SC	C	S	Male	Female
A (***Pst*** **I** ^**+**^ **/** ***Bgl*** **I** ^**−**^)	0	0	0	0	7	7	0
B (***Pst*** **I** ^**+**^ **/** ***Bgl*** **I** ^**+**^)	10	11	5	20	6	32	20
C (***Pst*** **I** ^**−**^ **/** ***Bgl*** **I** ^**+**^)	0	0	0	0	1	0	1
D (***Pst*** **I** ^**−**^ **/** ***Bgl*** **I** ^**−**^)	0	0	0	0	0	0	0
							

N = North; NC = North Central; SC = South Central; C = Central; S = South.

In the distribution of VZV genotypes among genders, we found that all the male samples had genotype A (*Pst*I^+^/*Bgl*I^−^). We did not find genotype A among females, and the males had higher number of B type than the females. One female sample had genotype C (*Pst*I^−^/*Bgl*I^+^) similar to oka genotype. All Indian samples exhibited maximum similarity among their compatriots (Table 2).

## DISCUSSION

Genotyping is a useful epidemiologic tool for many human herpes viruses although it has not generally led to good correlations with virus pathology (29–32). An ideal genotyping format permits reliable VZV genotyping using a single short amplicon, or, if necessary, a very limited number of amplicons. The VZV genome is extremely stable; all three genotypes of VZV differ in their DNA sequence by about 0.2% only. The low number of replicative cycles in the infected host (33) is thought to restrict opportunities for introducing mutations.

With the introduction of varicella vaccination by private hospitals in the State of Kuwait, a surveillance program for VZV strains occurring in case of varicella is needed. A number of genetic markers that can identify and distinguish wild-type VZV strains from the vaccine Oka strain have been studied previously, such as ORF 6 (*Alu*I), ORF 38 (*Pst*I), ORF 54 (*Bgl*I), ORF 62 (*Sma*I, VZV ORF 62 based genotyping *Nae*I, *BssH*II) (30, 34, 35). In this study, we confirmed that most of the genotypes A and B, the VZV wild-type strains can be distinguished from the Oka vaccine strain by using the *Pst*I marker in ORF 38. Genetic polymorphisms in ORFs 38 (*Pst*I) and 54 (*Bgl*I) have been used extensively in vaccine and epidemiological studies (30, 36, 37). The ORF 38–54 PCR-RFLP technique was able to discriminate between Wild-type VZV strains circulating among the Indian patients that are of the *Pst*I^+^/ *Bgl*I^+^ genotype, as opposed to the Oka vaccine strain, which has the *Pst*I^−^ /*Bgl*I^+^ genotype (17, 29). In this study, all isolates except one were wild type, with no Oka vaccine-associated markers.

Analysis based on ORF22 SNPs alone (17, 38) indicated that (*Pst*I^+^ *Bgl*I^−^) strains (A genotype by our method) constituted a uniform, single genotype and represented the dominant genotype in temperate climates. In contrast, the use of genotyping method 2 (analysis of SNPs in at least four to five amplicons from ORFs 1, 21, 50, 54, and 68) revealed that the E-type strains were subdivided into two distinct genotypes (18). Given the very limited number of genetic changes involved, it remains a possibility that these variant strains could have simply arisen through mutation. However, the recent analyses available in literature suggest the otherwise (23).

In this study, we have successfully differentiated circulating wild-type VZV strains in the State of Kuwait from that of the vaccine Oka strain with the ORF-38-54- based PCR-RFLP method. In addition, this method will also serve to identify and differentiate Oka-like wild-type strains that may possibly enter the circulation but are likely to be missed by less discriminatory methods (39).

In Europe, a significant fraction of isolated wild-type viruses belong to genotype A (*Pst*I^−^/ *Bgl*I^+^) (37). However this is clearly not the case in Kuwait where genotype B strains represented about 86% of the wild-type VZV samples obtained from clinical cases of varicella in Kuwait. All of these strains were *Pst*I^+^ / *Bgl*I^+^, consequently, ORF38/ORF54-based SNP analysis is a useful approach for discriminating vaccine from wild-type strains, since no genotype D (*Pst*I^−^ /*Bgl*I^−^) strains were found circulating in the samples tested in Kuwait.

Admittedly, we have so far examined only a small numbers of clinical samples that may or may not reflect all the VZV strains that are circulating throughout the State of Kuwait. Nonetheless, testing of additional clinical specimens should help to reinforce the validity of this method, particularly in specimens from countries where Oka-like strains may still be circulating. Protocols using this approach for diagnosing suspected chicken pox in clinical samples should be coupled with PCR, using primers specific for beta-globin gene DNA or other cellular markers to confirm that amplification conditions are optimal, thus minimizing false-negative results (40).

This is the first study carried out on genotyping VZV from clinical samples collected in the State of Kuwait. The present study extends the usefulness of PCR techniques as a diagnostic tool for the detection and differentiation of VZV DNA in clinical specimens.
